# A systematic revision of *Capparaceae* and *Cleomaceae* in Egypt: an evaluation of the generic delimitations of *Capparis* and *Cleome* using ecological and genetic diversity

**DOI:** 10.1186/s43141-020-00069-z

**Published:** 2020-10-06

**Authors:** Mohamed Abd. S. El zayat, Mahmoud El Sayd Ali, Mohamed Hamdy Amar

**Affiliations:** 1 Egyptian Deserts Gene Bank, Desert Research Center, Cairo, Egypt; 2 Department of Plant Ecology, Desert Research Center, Cairo, Egypt

**Keywords:** *Capparaceae*, *Cleomaceae*, Systematic revision, Genetic diversity, Ecological distribution

## Abstract

**Background:**

The *Capparaceae* family is commonly recognized as a caper, while *Cleomaceae* represents one of small flowering family within the order *Brassicales*. Earlier, *Cleomaceae* was included in the family *Capparaceae*; then, it was moved to a distinct family after DNA evidence. Variation in habits and a bewildering array of floral and fruit forms contributed to making *Capparaceae* a “trash-basket” family in which many unrelated plants were placed. Indeed, family *Capparaceae* and *Cleomaceae* are in clear need of more detailed systematic revision.

**Results:**

Here, in the present study, the morphological characteristics and the ecological distribution as well as the genetic diversity analysis among the twelve species of both *Capparaceae* and *Cleomaceae have been determined*. The genetic analysis has been checked using 15 ISSR, 30 SRAP, and 18 ISTR to assess the systematic knots between the two families. In order to detect the molecular phylogeny, a comparative analysis of the three markers was performed based on the exposure of discriminating capacity, efficiency, and phylogenetic heatmap. Our results indicated that there is a morphological and ecological variation between the two families. Moreover, the molecular analysis confirmed that ISTR followed by SRAP markers has superior discriminating capacity for describing the genetic diversity and is able to simultaneously distinguish many polymorphic markers per reaction. Indeed, both the PCA and HCA data have drawn a successful annotation relationship in *Capparacea*e and *Cleome* species to evaluate whether the specific group sort individual or overlap groups.

**Conclusion:**

The outcomes of the morphological and ecological characterization along with the genetic diversity indicated an insight solution thorny interspecies in *Cleome* and *Gynandropsis* genera as a distinct family (*Cleomaceae*) and the other genera (*Capparis*, *Cadaba*, *Boscia*, and *Maerua*) as *Capparaceae*. Finally, we recommended further studies to elucidate the systematic position of *Dipterygium glaucum.*

## Background

The flora of Egypt is among the richest within the Arab countries and comprises very vital genetic resources of medicinal, fodder, and fiber plants. Currently, plant diversity is under threat as never before. In agriculture, the broad selection of a few developed varieties has reduced the genetic base of the most essential food crops, and it has added to the withdrawal of hundreds of landraces [1]. The complicated relationship between the three closely related families, *Capparaceae*, *Cleomaceae*, and *Brassicaceae*, has been extensively studied since the appearance of the theory of alternation of generation by Hofmeister [2]. Hutchinson [3] in his phylogenetic work differentiated between *Capparaceae* and *Brassicaceae* according to their morphological features to two separate families. Shifting of some species between the two families is common, like *Dipterygium glaucum* has been treated as genera of *Brassicaceae* [4], then moved to *Capparaceae* as a subfamily: *Dipterigpideae* [5]. Tackholm [6] classifies *Dipterygium* as a species of *Cruciferae*, while Hedge et al. [7] classify it as a member of *Capparaceae*. This assumption has been reinforced by Boulos [8].

In the Egyptian flora, the set of *Capparaceae* plus *Cleomaceae* includes seven genera, twenty-two species, and four varieties with a wide range of ecological and geographical distribution [8]. They differ considerably in their life forms from trees (e.g., *Boscia angustifolia*) or shrubs (e.g., *Capparis cartilaginea*) to annual (e.g., *Gynandropsis gynandra*) or perennial herbs (e.g., *Cleome amblyocarpa*). The Egyptian taxa of *Capparaceae* refer to the xerophytic communities [9, 10], without *Gynandropsis gynandra* that is a common weed of the arable fields [11]. The taxonomic approach of the family in Egypt is interested only on seed morphology [12], leaf anatomy [13], and pollen morphology [14]. The systematic review of the natural species of *Capparaceae* (except *Cleome*) announced the unclear occurrence of *Boscia angustifolia*, while *Capparis spinosa* is described by three varieties, viz., *deserti*, *spinosa*, and *inermis* [15]. Spilt from *Cleomaceae* may be unreasonable since complications are met in selecting the genera. Accurate qualified information on fruit construction and gynoecium is obscure or non-present. Therefore, the taxonomic relationships between *Capparaceae* and *Cleomaceae* are still at discussion. Tackholm [6] distinguished among the two families concurring to gland formation, fruit type, and development of gynophores, whereas Zohary [16] included the exciting genus *Cleome* in the subfamily *Cleomoideae* of *Capparaceae*. The *Capparaceae* in Boulos [8], however, involved together *Cleomaceae* and *Capparaceae*. On the species level, Tackholm [6] identified eight species of *Capparis*, while Boulos [8] divided the genus as three species and four varieties. Though flower and fruit types have been shown very beneficial in the description and definition of the genera and species, there are situations in which these tools are not potential for the study as in *Capparaceae*. The subject of the reproductive characteristics of this group is uncertain for various purposes, among others; the challenge of maintaining the flowers in some genera as in *Capparis* [17] the unusual variability in their dimension and form at the individual level species [18] and several long-lived tropical plant flowers are uncommon and different [19]. Therefore, there is a great necessity to distinguish and order the wild plants utilizing morphological and molecular tools. Hence, the investigation of molecular information is the main outcome in our opinion of plant phylogeny and systematic, from the low joints dividing of the main plant groups to varieties and populations [20]. Molecular identification represents an effective instrument for genome analysis and allows the linkage of heritage traits attached to genomic divergence. Presently, these genomic tools are valuable basics for knowledge and improving resource for understanding and developing various frequent sequence approaches such as microsatellites and retrotransposon loci. Within PCR-based approaches, inter-simple sequence repeats (ISSRs) became to be addressed development of minisatellite DNA for classification of varieties or species and population genetic structure, with high efficiency, stability, low cost, and simple operation [21, 22]. Previous research of ISSR studies on several wild plant species, involving endangered and rare genus, have elucidate the hypervariable type of microsatellite loci and their inherent utility in species identification and inhabitant’s diversity [23–25], while sequence-related amplified polymorphism (SRAP) technique, which favorably aims regions including promoters and exonic sequences [26], have several performances, containing the use for a rather minor quantity of DNA, and high levels of polymorphism amplification targeting an open reading frames (ORFs) in many plant species [27].

In regard to inverse sequence-tagged repeat (ISTR), it is a retrotransposon-based marker [28] which has been viewed in most form of the organism, is widespread in distribution, effective, and plentiful in eukaryotic genomes [29, 30]. Therefore, ISTR markers are co-dominant markers which can define the wild flora and phylogeny at a subgenus level [31]. The three molecular markers recorded beyond are co-dominant or dominant inheritance, and the collectively utilized could be farther beneficial to identify diverse portions of the genome [32]. Consequently, comparison is necessary to determine the marker sensitivity and appropriateness for the topic being studied [33].

The current investigation was aimed to provide a broad description of the morphological, ecological distribution attributes, and connected species along with three selected molecular markers of 12 representing species of *Capparaceae* and *Cleomaceae*. In detail, first, family *Capparaceae* is in clear necessity of more detailed morphological, ecological, and molecular study. Modulation inhabits and a confusing display of floral and fruit profiles provided to forming *Capparaceae* a “trash-basket” family in which several discrete plants were ordered. Second is to evaluate the discrimination capacity and the performance of the three marker system involving ISSR, SRAP, and ISTR and, finally, to infer the taxonomic questions and species borders treating the genetic diversity of Egyptian *Capparaceae and Cleomaceae* germplasm. To date, there has been no announcement concerning the performance and effectiveness of ISSR, SRAP, and ISTR markers in Egyptian *Capparaceae* and *Cleomaceae* species.

## Methods

### Plant materials

**Fig. 1:**
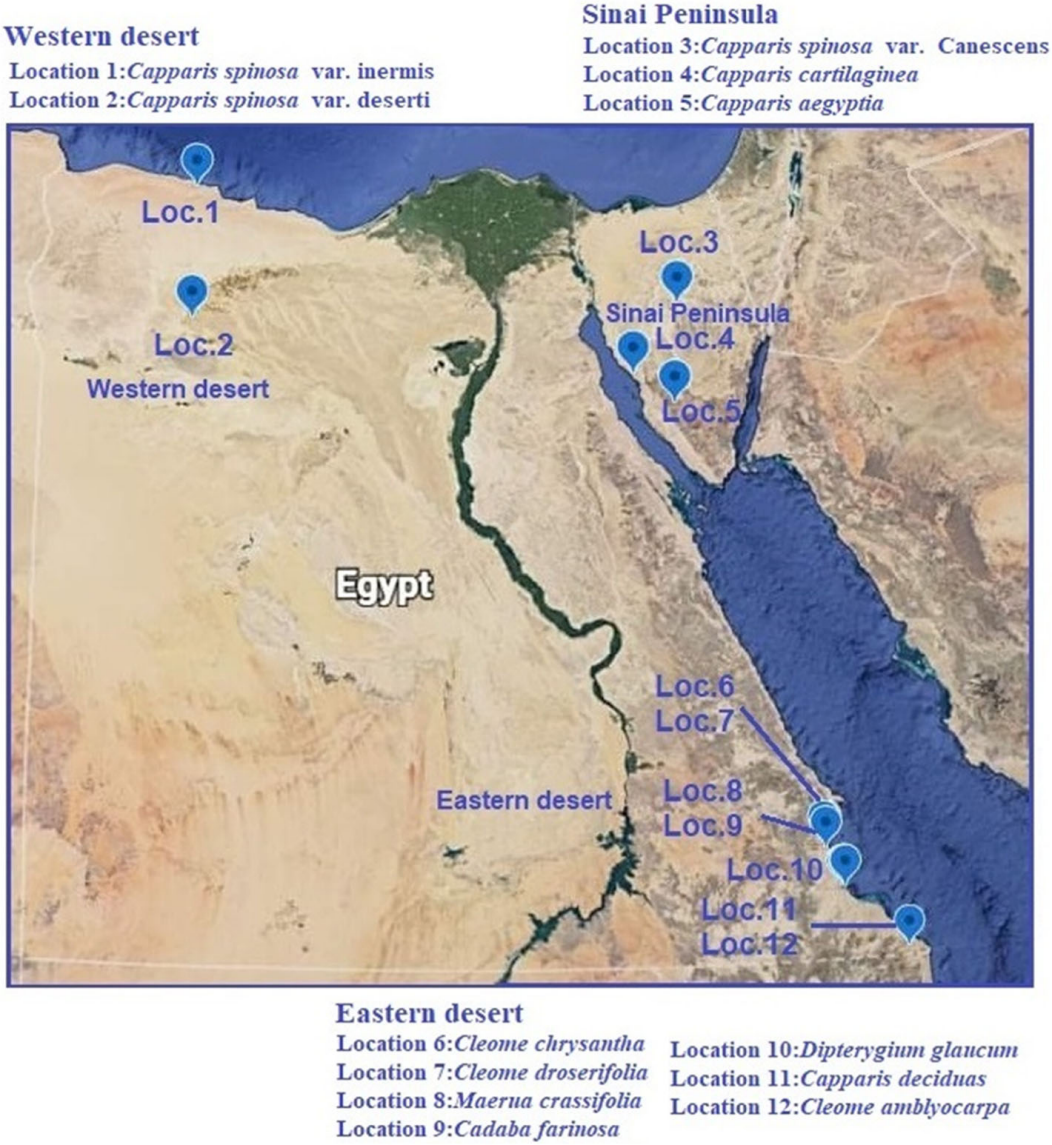
Map showing the distributions and locations of 12 *Capparaceae* species within Western, Eastern desert, and Sinai Peninsula in Egypt

A total of twelve plant samples of *Capparaceae* and *Cleomaceae* were collected from Sinai Peninsula (three plants), Western desert (two plants), and Eastern desert (seven plants) (Fig. 1) (Table 1). Associate species were recorded, and identification was performed according to Boulos [8, 11]. Plant density and cover were assessed by the random plot and line intercept methods. A hierarchical dichotomous analysis (TWINSPAN) was used to classify plant species due to their density by PAST 3.14 [34].

**Table 1:** A list of selected *Capparaceae* and *Cleomaceae* species used in this study with ISSR, SRAP, and ISTR primers showing their locations and co-ordinates

No.	Species	Latitude	Longitude	Altitude/m	Location
1	*Capparis spinosa* var. *inermis* Turra.	31 19 21	27 04 20.4	85	Western desert
2	*Capparis spinosa* var. *deserti* Zohary.	29 44 50.5	25 47 13.2	153	Western desert
3	*Capparis spinosa* var. *Canescens* Coss.	29 54 05.6	33 42 56.1	402	Sinai Peninsula
4	*Capparis cartilaginea* Decne.	28 42 48.1	33 39 45.7	696	Sinai Peninsula
5	*Capparis aegyptia* Lam.	29 04 58.2	33 05 55.2	23	Sinai Peninsula
6	*Cleome chrysantha* Decne.	23 25 20.7	34 52 30.5	291	Eastern desert
7	*Cleome droserifolia* Forssk.	23 29 48.8	35 10 51	329	Eastern desert
8	*Maerua crassifolia* Forssk.	22 55 18.8	36 21 15.8	22	Eastern desert
9	*Cadaba farinosa* Forssk.	22 55 18.8	36 21 15.8	22	Eastern desert
10	*Dipterygium glaucum* Decne.	23 24 17.7	35 29 52	21	Eastern desert
11	*Capparis decidua* (Forssk.) Edgew.	22 55 18.8	36 21 15.8	22	Eastern desert
12	*Cleome amblyocarpa* Barratte & Murb.	22 05 24	36 37 35	148	Eastern desert

### DNA extraction

A fresh leaf of *Capparaceae* and *Cleomaceae* samples was used to extract the total genomic DNA according to the manufacturer’s protocol using plant DNA purification mini kit (Thermo Scientific GeneJET kit, K0791, USA). In each species, three to five replicate DNAs were utilized. However, the concentration and quality of the DNA samples were verified in a Quawell Q5000 UV-Vis spectrophotometer (V2.1.4, USA); then, the DNA was diluted to 50 ng/μl for use in ISSR, SRAP, and ISTR assay. Both the stock and diluted portions were kept at − 20 °C.

#### ISSR analysis

The ISSR-PCR amplification was prepared according to the earlier method demonstrated by Sankar and Moore [35]. The amplification reaction of ISSR analysis was done in a total volume of 25 μl on a Sure Cycler 8800 Thermal Cycler from Agilent Technologies. The reaction combination of 25 μl involved 3.5 μl of Green PCR buffer, 1 μM of each primer, 0.5 μM of dNTPs (10 mM each) (Thermo Fisher Scientific), 1 unit of taq DNA polymerase (5 U/μL) (Thermo Fisher Scientific), and 40 ng DNA template. The PCR program was as follows, denaturation (one cycle) in 94 °C for 2 min, followed by 40 cycles as follows: 94 °C for 30 s, 44 °C for 45 s, 72 °C for 1 min and 30 s, and finally one cycle extension at 72 °C for 10 min, and 4 °C (infinitive). The amplified products were separated on 1.2% agarose gel by electrophoresis. A 100 bp DNA ladder (GeneRuler plus, Thermo Scientific, SM0321) was utilized as the molecular guideline to verify the competent ISSR markers. The gels were stained in ethidium bromide (0.5 μg/ml), and the amplicons were pictured below UV light using the Gel Doc XR system (Bio-rad, America).

#### SRAP analysis

The SRAP analysis was presented as illustrated by Li and Quiros [26]. SRAP primer combinations were tested using 30 various combinations which employed utilizing seven reverse and nine forward primers applied (Table 2). Every PCR reaction mix of 25 μl included 3.5 μl of green PCR buffer, 0.3 μM of each primer, 200 μM of dNTPs, 1 unit of taq DNA polymerase, 30 ng of genomic DNA, and deionized water up to 25 μl. PCR cycling program comprised 4 min of denaturing at 94 °C, five cycles of three steps: 1 min of denaturing at 94 °C, 1 min of annealing at 35 °C, and 1 min of elongation at 72 °C. In the next 35 cycles, the annealing temperature was increased to 50 °C, and for an extension, one cycle of 7 min at 72 °C. GeneRuler 50 bp Plus DNA ladder (Thermo Scientific, SM0371) was utilized as a molecular guideline to verify the accurate SRAP markers.

**Table 2:** List of ISSR, SRAP and ISTR primer combinations obtained from the current investigation

Primer name		Sequence	Primer name		Sequence	
HB1		(CAA)5	HB 12		(CAC)3 GC	
HB 2		(CAG) 5	HB 13		(GAG)3 GC	
HB 4		(GACA) 4	HB 15		(GTG) 3 GC	
HB 8		(GA)6 GG	807		(AG)8 T	
HB 9		(GT) 6 GG	814		(CT) 8TG	
HB 10		(GA) 6 CC	844A		(CT)8 AC	
HB 11		(GT) 6 CC	844B		(CT) 8 GC	
17899B		(CA) 6 GG				
Marker type	Marker Name	Forward primer	Reverse primer	Marker Name	Forward primer	Reverse primer
SRAP	Em 1R/DN 6 F	GACTGCGTACGAATTAAT	/TGAGTCCAAACCGGTAA	Em 10 R/DN 8 F	GACTGCGTACGAATTCAT	TGAGTCCAAACCGGTGC
	Em 1R/DN 7 F	GACTGCGTACGAATTAAT	TGAGTCCAAACCGGTCC	Em 10 R/DN 9 F	GACTGCGTACGAATTCAT	TGAGTCCAAACCGGTCA
	Em 1R/DN 8 F	GACTGCGTACGAATTAAT	TGAGTCCAAACCGGTGC	Em 10 R/DN 10 F	GACTGCGTACGAATTCAT	TGAGTCCAAACCGGGCT
	Em 1R/DN 9 F	GACTGCGTACGAATTAAT	TGAGTCCAAACCGGTCA	Em 10 R/DN 12 F	GACTGCGTACGAATTCAT	TGAGTCCAAACCGGTGT
	Em 1R/DN 10 F	GACTGCGTACGAATTAAT	TGAGTCCAAACCGGGCT	Em 12 R/DN 11 F	GACTGCGTACGAATTCTC	TGAGTCCAAACCGGTAG
	Em 1R/DN 11 F	GACTGCGTACGAATTAAT	TGAGTCCAAACCGGTAG	Em 15 R/DN 6 F	GACTGCGTACGAATTGTC	TGAGTCCAAACCGGTAA
	Em 1R/DN 12 F	GACTGCGTACGAATTAAT	TGAGTCCAAACCGGTGT	Em 15 R/DN 8 F	GACTGCGTACGAATTGTC	TGAGTCCAAACCGGTGC
	Em 1R/DN 6 F	GACTGCGTACGAATTAAT	TGAGTCCAAACCGGTAA	Em 15 R/DN 9 F	GACTGCGTACGAATTGTC	TGAGTCCAAACCGGTCA
	Em 1R/DN 7 F	GACTGCGTACGAATTAAT	TGAGTCCAAACCGGTCC	Em 15 R/DN 10 F	GACTGCGTACGAATTGTC	TGAGTCCAAACCGGGCT
	Em 1R/DN 8 F	GACTGCGTACGAATTAAT	TGAGTCCAAACCGGTGC	Em 15 R/DN 12 F	GACTGCGTACGAATTGTC	TGAGTCCAAACCGGTGT
	Em 1R/DN 9 F	GACTGCGTACGAATTAAT	TGAGTCCAAACCGGTCA	Em 18 R/DN 12 F	GACTGCGTACGAATTGAG	TGAGTCCAAACCGGTGT
	Em 1R/DN 10 F	GACTGCGTACGAATTAAT	TGAGTCCAAACCGGGCT	Em 20 R/DN 6 F	GACTGCGTACGAATTGCC	TGAGTCCAAACCGGTAA
	Em 1R/DN 11 F	GACTGCGTACGAATTAAT	TGAGTCCAAACCGGTAG	Em 20 R/DN 7 F	GACTGCGTACGAATTGCC	TGAGTCCAAACCGGTCC
	Em 1R/DN 12 F	GACTGCGTACGAATTAAT	TGAGTCCAAACCGGTGT	Em 20 R/DN 8 F	GACTGCGTACGAATTGCC	TGAGTCCAAACCGGTGC
	Em 6 R/DN 7 F	GACTGCGTACGAATTGCA	TGAGTCCAAACCGGTCC	Em 20 R/DN 9 F	GACTGCGTACGAATTGCC	TGAGTCCAAACCGGTCA
	Em 8 R/DN 8 F	GACTGCGTACGAATTCTG	TGAGTCCAAACCGGTGC	Em 20 R/DN 10 F	GACTGCGTACGAATTGCC	TGAGTCCAAACCGGGCT
	Em 9 R/DN 9 F	GACTGCGTACGAATTCAG	TGAGTCCAAACCGGTCA	Em 20 R/DN 11 F	GACTGCGTACGAATTGCC	TGAGTCCAAACCGGTAG
	Em 10 R/DN 6 F	GACTGCGTACGAATTCAT	TGAGTCCAAACCGGTAA	Em 20 R/DN 12 F	GACTGCGTACGAATTGCC	TGAGTCCAAACCGGTGT
	Em 10 R/DN 7 F	GACTGCGTACGAATTCAT	TGAGTCCAAACCGGTCC			
ISTR	F1/B2	AGGAGGTGAATACCTTAG	GGATATCCTATGAATCAAGC	F3/B2	GTCGACATGCCATCTTTC	GGATATCCTATGAATCAAGC
	F1/B3	AGGAGGTGAATACCTTAG	ATTCCCATCTGCACCAAT	F3/B3	GTCGACATGCCATCTTTC	ATTCCCATCTGCACCAAT
	F3/B5	GTCGACATGCCATCTTTC	CTTCTGTGAAAGTCCTAG	F3/B6	GTCGACATGCCATCTTTC	ATATATGGACTTAAGCAAGCA
	F3/B8	GTCGACATGCCATCTTTC	CCTCCTTATTGGGAATGATAT	F7/B3	TGCTAGGACTTTCACAGA	ATTCCCATCTGCACCAAT
	F3/B10	GTCGACATGCCATCTTTC	GACCCTTTTGAAAACACATG	F7/B6	TGCTAGGACTTTCACAGA	ATATATGGACTTAAGCAAGCA
	F5/B7	ATATATGGACTTAAGCAAGC	GGAATATCATTCCCAATAAG	F9/B2	ATATGGACTTAAGCAAGCCA	GGATATCCTATGAATCAAGC
	F5/B8	ATATATGGACTTAAGCAAGC	CCTCCTTATTGGGAATGATAT	F9/B3	ATATGGACTTAAGCAAGCCA	ATTCCCATCTGCACCAAT
	F10/B6	GGAATATCATTCCCAATAAG	ATATATGGACTTAAGCAAGCA	F9/B6	ATATGGACTTAAGCAAGCCA	ATATATGGACTTAAGCAAGCA
	F1/B8	AGGAGGTGAATACCTTAG	CCTCCTTATTGGGAATGATAT	F9/B10	ATATGGACTTAAGCAAGCCA	GACCCTTTTGAAAACACATG

#### ISTR analysis

ISTR assessment was conducted following Aga and Bryngelsson [36]. ISTR primer combinations were primarily examined using a total of 70 primer combinations from seven reverse and ten forward primers. Within all primers screened, only 24 ISTR combinations were picked for advanced analysis (Table 2). Each PCR included a reaction mix of 3.5 μl of green PCR buffer, 200 μM of dNTPs, 0.3 μM of each primer, 50 ng of genomic DNA, 1 unit of taq DNA polymerase, and finally deionized water up to 25 μl. PCR amplification performed involved of 1 cycle at 95 °C, 3 min; 40 cycles of 94 °C, 30 s; 45 °C, 30 s; 72 °C, 2 min; 1 cycle at 72 °C, 10 min; and 4 °C for infinitive. However, amplification products were separated and visualized subsequent the same procedure described for ISSR.

### Data analysis

All clearly detectable ISSR, SRAP, and ISTR products were counted as band absence (0) and presence (1) using the Bio-Rad Gel Doc™ XR+ imaging analysis system with Image Lab™ (USA), and adjusted manually as necessary and collected onto a data matrix. However, the evaluations of the marker efficiency, level of polymorphism, discriminating capacity, and informativeness of the three marker profiles were calculated according to the indices of Powell et al. [37].

To measure the effectiveness of the three marker systems, polymorphic information content (PIC) was analyzed using the following formula of Roldán-Ruiz et al. [38] PIC = 2*fi*(1 − *fi*), where *fi* is the frequency of the amplified allele and 1 – *fi* is the frequency of the null allele. While heterozygosity per locus was determined according to the formula: *He* = 1 − *p*2 − *q*2, where *p*2 = *fi*. Meanwhile, the average heterozygosity per marker was evaluated based on *Hav* = ∑(*He*/*L*), where *L* = total of detected bands. The multiplex ratio was calculated as *MR* = *L*/*T*, where *T* = the total number of primer combinations. The marker index (MI) was achieved by developing the average heterozygosity by the multiplex ratio: *MI* = *Hav X MR*.

To gain accurate perspectives on genetic diversity analyses among the *Capparaceae* and *Cleomaceae* germplasm, a graphic demonstration of principal coordinates analysis (PCA) and the heatmap cluster analysis (HCA) was provided an explanation to demonstrate the multidimensional genetic relationship and its split among species using ClustVis web tool for visualizing clustering of multivariate data [39].

## Results

### Morphological basis of *Capparaceae* taxonomy

**Fig. 2:**
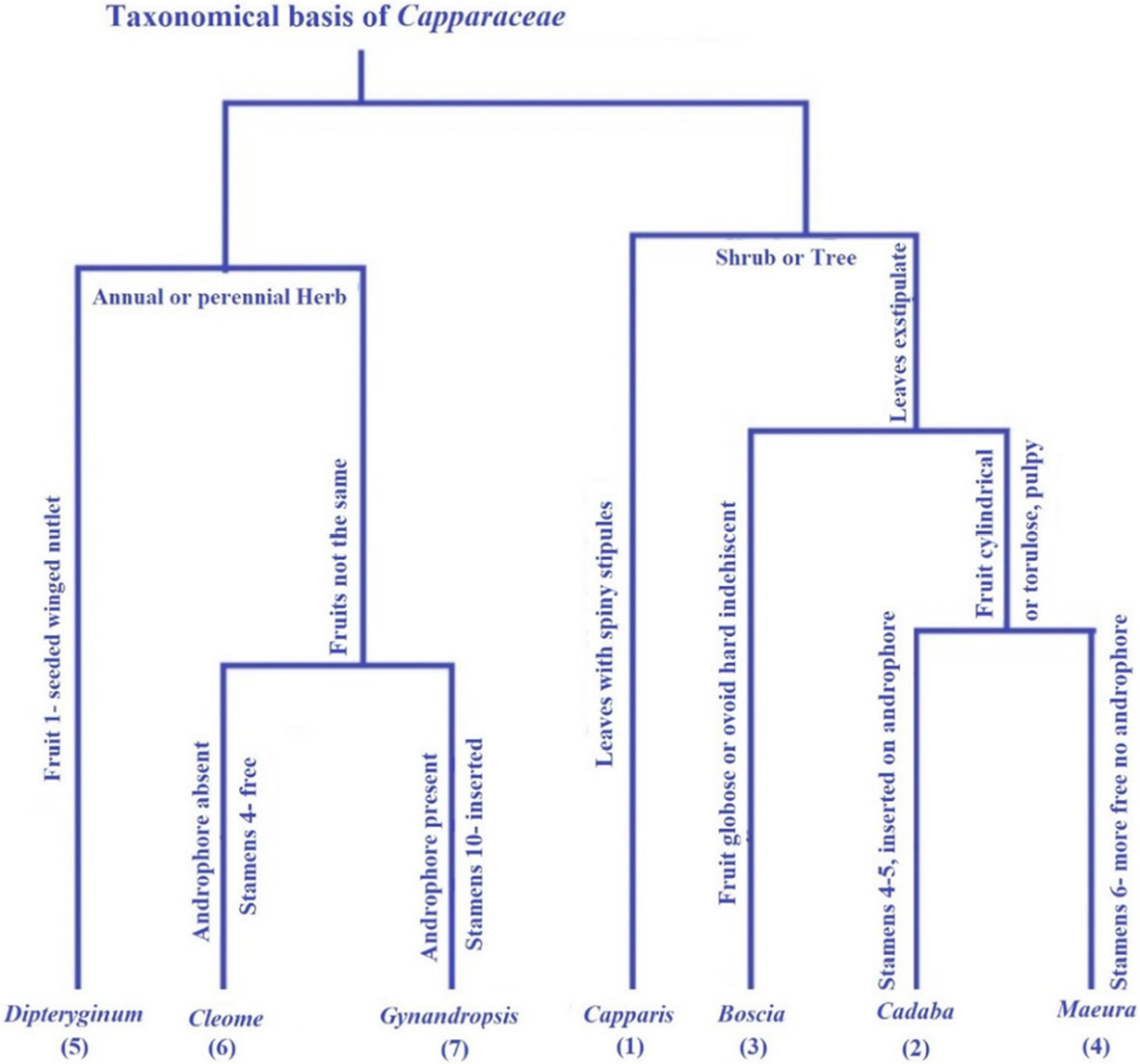
Morphological basis of *Capparaceae* taxonomy

In the present scenario, the main differences between *Capparis* and *Cleome* are growth habit and life span, as presented in Fig. 2. All individuals of *Capparaceae* are trees or shrubs, whereas species of *Cleome* are annual or perennial herbs. According to the type of fruits of the family, *Cleomaceae* were divided into *Dipterygium* (fruits one-seeded), *Gynandropsis*, and *Cleome* (fruits contains much seed), and then separated according to the number of stamens and presence or absence of androphore. On the other hand, family *Capparaceae* is classified into four species due to the presence of a stipule spine (*Capparis*) and the absence of a stipule (*Maeura*, *Cadaba*, and *Boscia*). *Boscia* species leave their group due to its fruit type (hard indehiscent). Recently, both *Maeura* and *Cadaba* are isolated according to the number of stamens and their contact with the androphore.

### Distribution and habitat classification of *Capparaceae* and *Cleomaceae* species

**Fig. 3:**
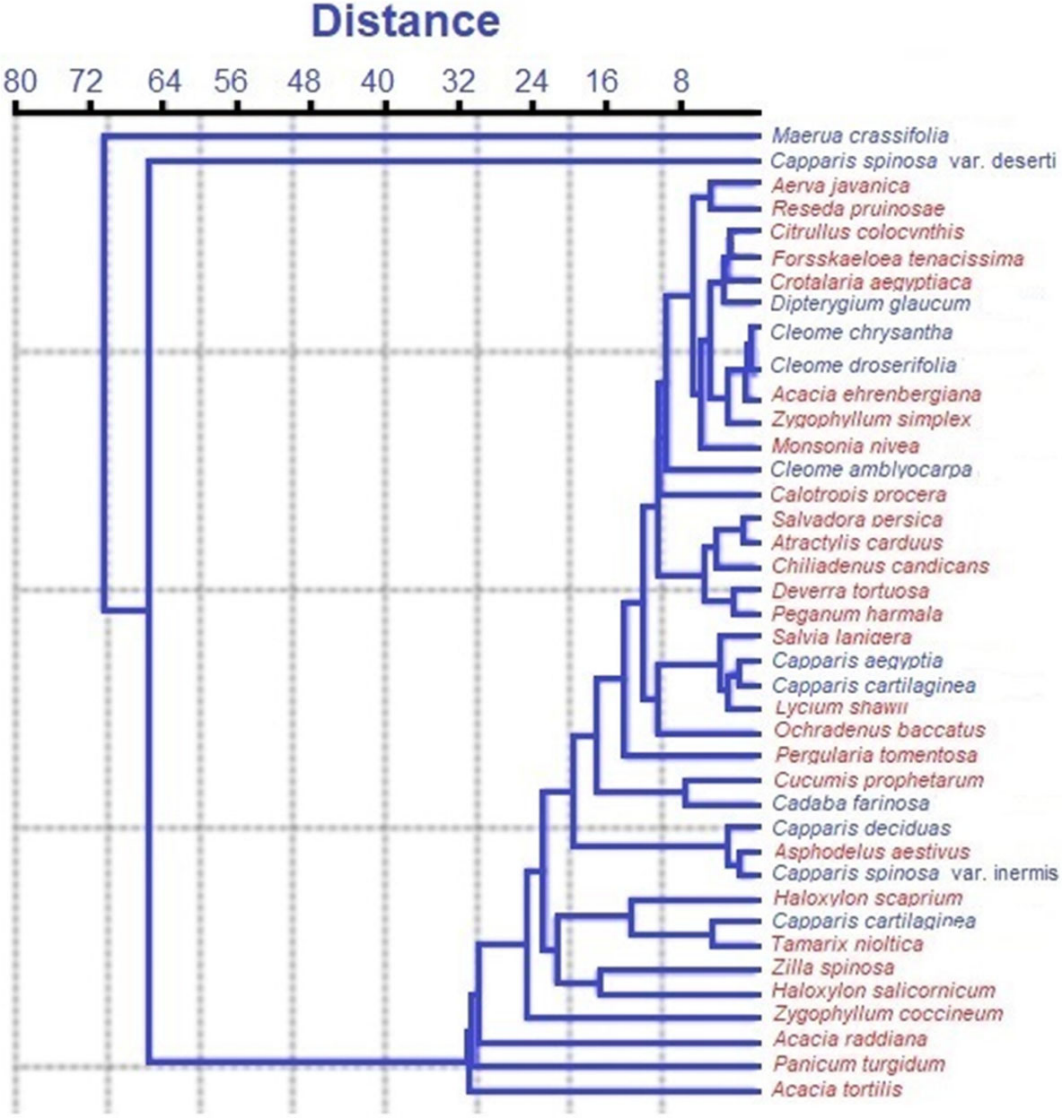
The tree represents the classification of the twelve selected *Capparaceae* and *Cleomaceae* species of the study, in blue, together with the 29 accompanying species in red

According to vegetation density and cover, classification of the twelve targeted species and 29 associates are mainly divided according to species distribution, habitat, and locations to six vegetation groups. The first group comprises of *Maerua crassifolia* and *Capparis deserti* inhabiting the sandy formation of the southern wadis of the Eastern desert and Siwa oasis, respectively. Moreover, they are tending to form pure communities with high density and cover. The second group is formed of *Cleome* and *Dipterygium*, and they are collected from the main channel of Wadi Abrq, Shalatein area. The third group involves of *Capparis spinosa* var. *canescens* and *C. aegyptia*, and they are similar in habitat; both of them are hanging between the rock fissures at south Sinai. While the fourth group (*Cadaba farinosa* and *Capparis decidua*) is distributed on the sandy formation of red sea wadis, the fifth group is represented with *Capparis spinosa* var*. inermis*; this species is restricted to the maritime cliffs and rocky ridges at the western Mediterranean section. However, the last group is occupied with the biggest *Capparis* species (*Capparis cartilaginea*) (Fig. 3).

### Comparison of polymorphic levels and informativeness obtained with ISSR, SRAP, and ISTR markers

**Fig. 4:**
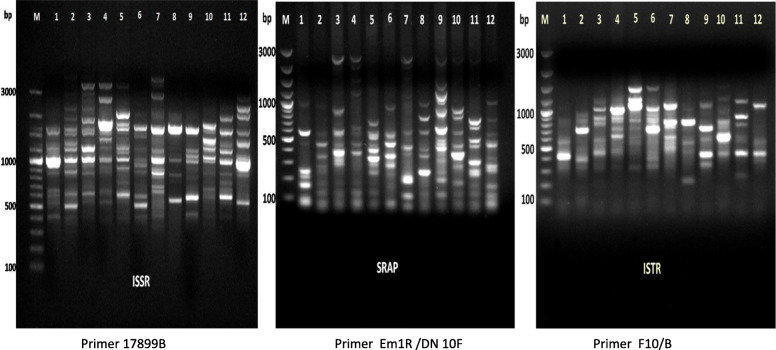
Observed the ISSR (**a**), SRAP (**b**), and ISTR (**c**) profiles of 12 *Capparaceae* species

**Fig. 5:**
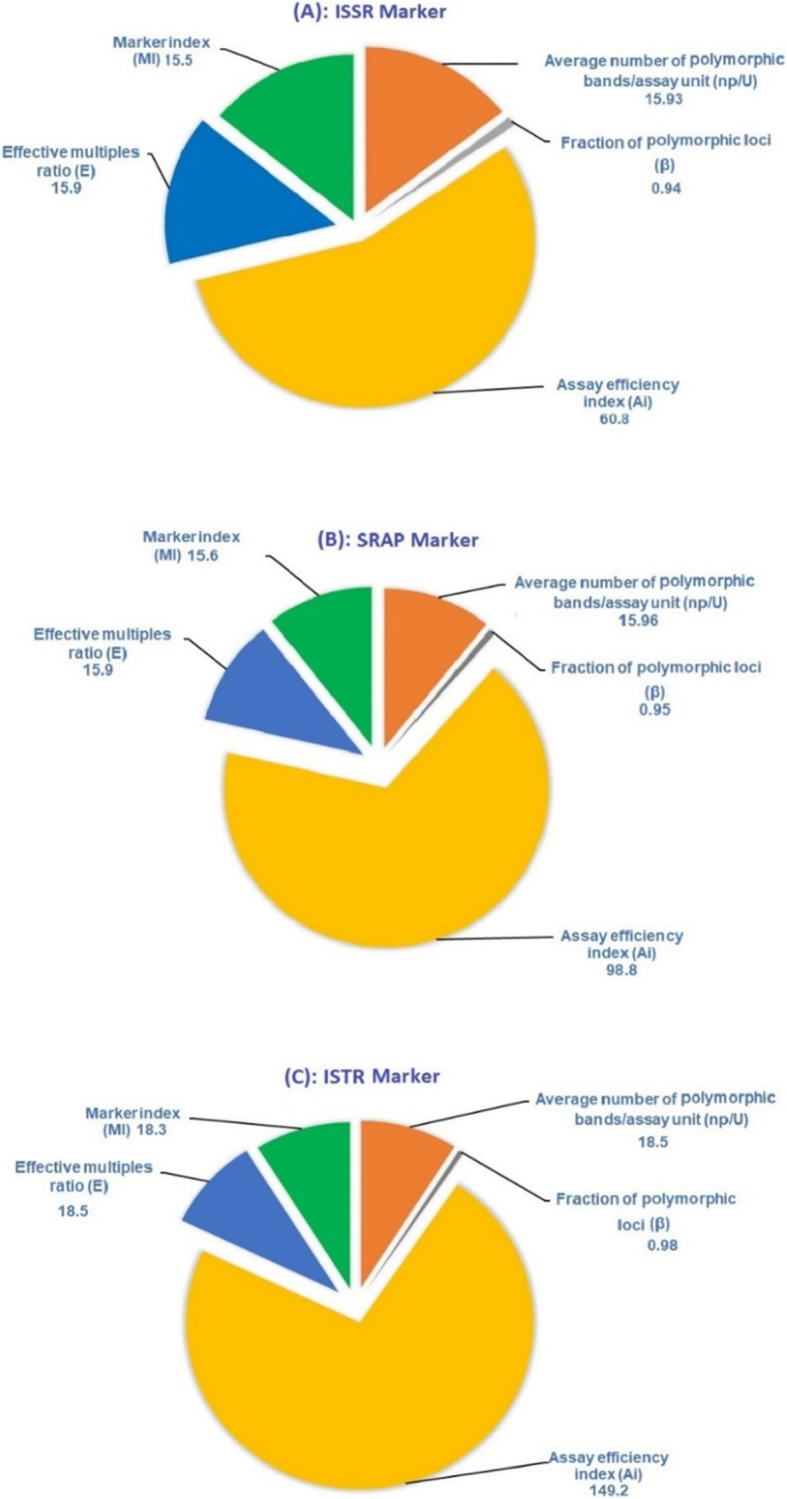
Observed the comparison information obtained and the discriminating capacity of **a** ISSR, **b** SRAP, and **c** ISTR profile among 12 *Capparaceae* species

In the present investigation, the levels of polymorphism of ISSR, SRAP, and ISTR markers and the index associating their informativeness are described in Table 3 and Fig. 4. All markers used pointed out to be helpful implements for the discovery of polymorphism and evaluating genetic diversity in *Capparaceae* and *Cleome* genotypes, but the level of sensitivity varied on the method applied. We primarily tested 20 of ISSR primers and 49 and 70 combinations of SRAP and ISTR primers among the twelve *Capparaceae* and *Cleome* species, respectively. Among all, only 15 ISSR, 30 SRAP, and 18 ISTR primers exhibited significant levels of polymorphism as shown in Table 3 and Fig. 4a–c. The total number of bands recorded for SRAP was almost high with 503 bands, followed by 337 and 252 bands for ISTR and ISSR markers, respectively. However, the total numbers of polymorphic bands (*p*) were ranged from 479 for SRAP, 333 for ISTR, to 239 for ISSR markers. On behalf of the total number of effective alleles (*Ne*), it was correlated significantly with the total number of bands (*L*) and the total numbers of polymorphic bands (*p*). Additionally, the average number of polymorphic bands/assay unit (*np/U*) was relatively high for ISTR being 18.5 with an intermediate value of 15.96 and 15.93 for SRAP and ISSR, respectively. Meanwhile, the PIC value for ISSR, SRAP, and ISTR marker system was almost parallel and relatively high being, 0.97, 0.98, and 0.99, respectively. Here, the present result showed that the ISTR marker was the most powerful marker in several detected parameters and PIC values. A comparative summary of the discriminating capacity of ISSR, SRAP, and ISTR markers are summarized in Table 3 and Fig. 5. On average, the three factors, assay efficiency index (*Ai*), effective multiples ratio (*E*), and marker index (*MI*), presented higher in ISTR marker, highlighting the notable characteristics of this marker compared to SRAP and ISSR (2.4×, 1.2× and 1.2× respectively).This certainly is due to the highest value of the assay efficiency index for the ISTR marker, inferring that ISTR has a higher discriminating capacity for counting the genetic diversity and can concurrently discover many polymorphic markers per reaction. Although the variances in some of the diversity statistics, these outcomes reveal that ISTR following by SRAP markers can be applied to assess the level of polymorphism in *Capparaceae* and *Cleome* species.

**Table 3:** Summary statistics of the information obtained with and discriminating capacity of ISSR, SRAP, and ISTR markers within twelve *Capparaceae* and *Cleomaceae* species

Marker efficiency	ISSR	SRAP	ISTR
Number of assay units (*N*)	15	30	18
Total number of bands (*L*)	252	503	337
Polymorphic bands (*p*)	239	479	333
Number of loci/assay unit (nu)	16.8	16.7	18.7
Total number of effective alleles (Ne)	912	2964	2687
Average number of polymorphic bands/assay unit(*np*/*U*)	15.93	15.96	18.5
Polymorphic information content (PIC)	0.97	0.98	0.99
Fraction of polymorphic loci (*β*)	0.94	0.95	0.98
Assay efficiency index (Ai)	60.8	98.8	149.2
Effective multiples ratio (*E*)	15.9	15.9	18.5
Marker index (MI)	15.5	15.6	18.3

### Diversity analyses and phylogenetic heatmap

**Fig. 6:**
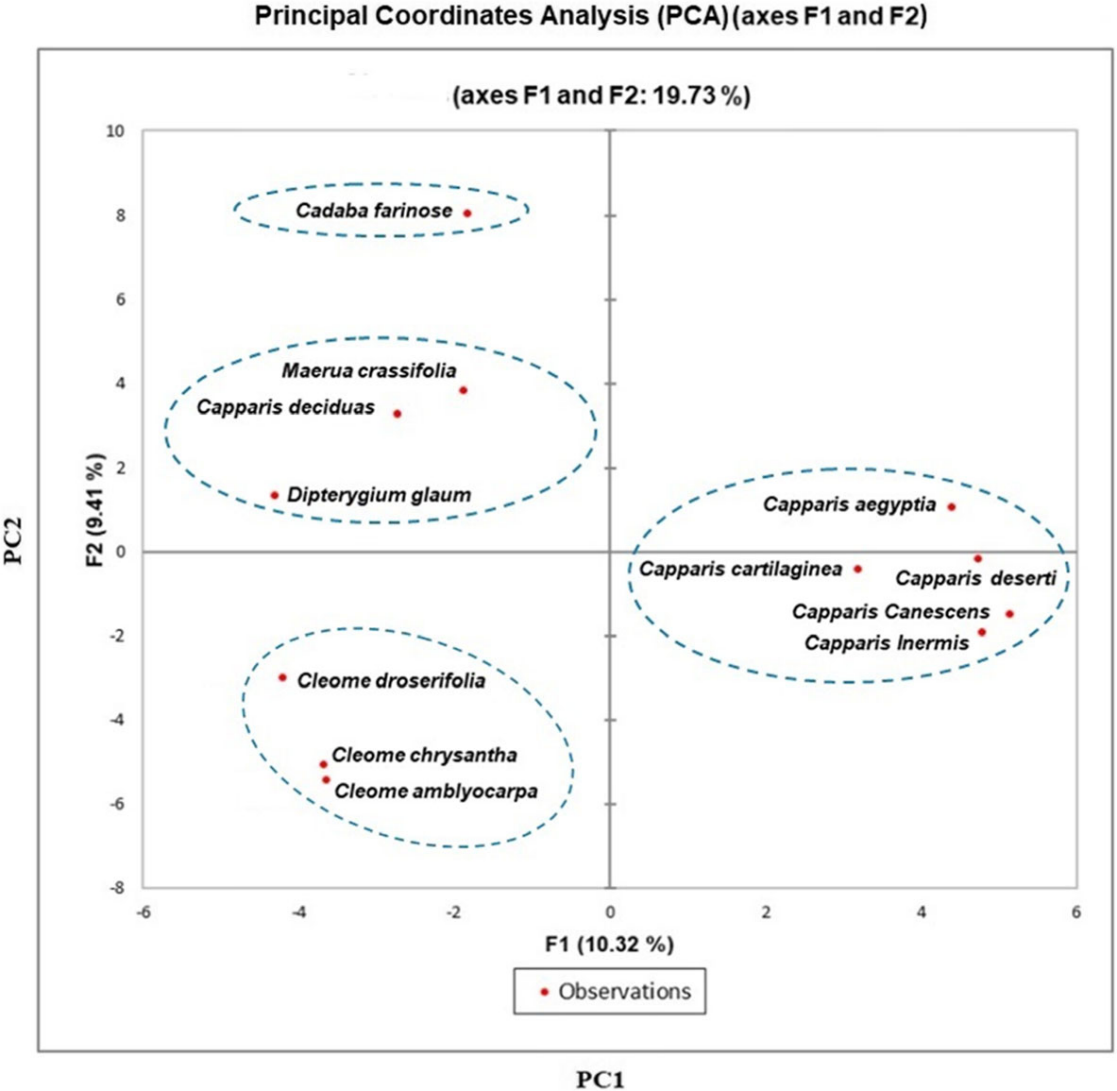
Schematic representation the principal coordinates analysis of 12 *Capparaceae* species based on ISSR, SRAP and ISTR markers. PC1and PC2 refer to the first and second principal component, respectively

Here, we present a graphic demonstration of both PCA and HCA, utilizing variable information matrix as input, wherever numerous features of ISSR, SRAP, and ISTR marker data are assessed in several observations. The overall PCA plot data for the three marker profile as shown in Fig. 6 created four relatively clustered groups, with a total of 19.73% of the molecular variance (PC1—10.32%, PC2—9.41%). Cluster I compressed *Capparis spinosa inermis*, *Capparis spinosa* var*. canescens*, *Capparis spinosa deserti*, *Capparis cartilaginea*, and *Capparis aegyptia* with a closer relationship than other groups. Moreover, cluster II assembled *Cleome droserifolia*, *Cleome chrysantha*, and *Cleome amblyocarpa* in a particular group, while *Maerua crassifolia*, *Capparis decidua*, and *Dipterygium glaucum* places jointly as cluster III. However, *Cadaba farinosa* was separated individually as out-group species.

**Fig. 7:**
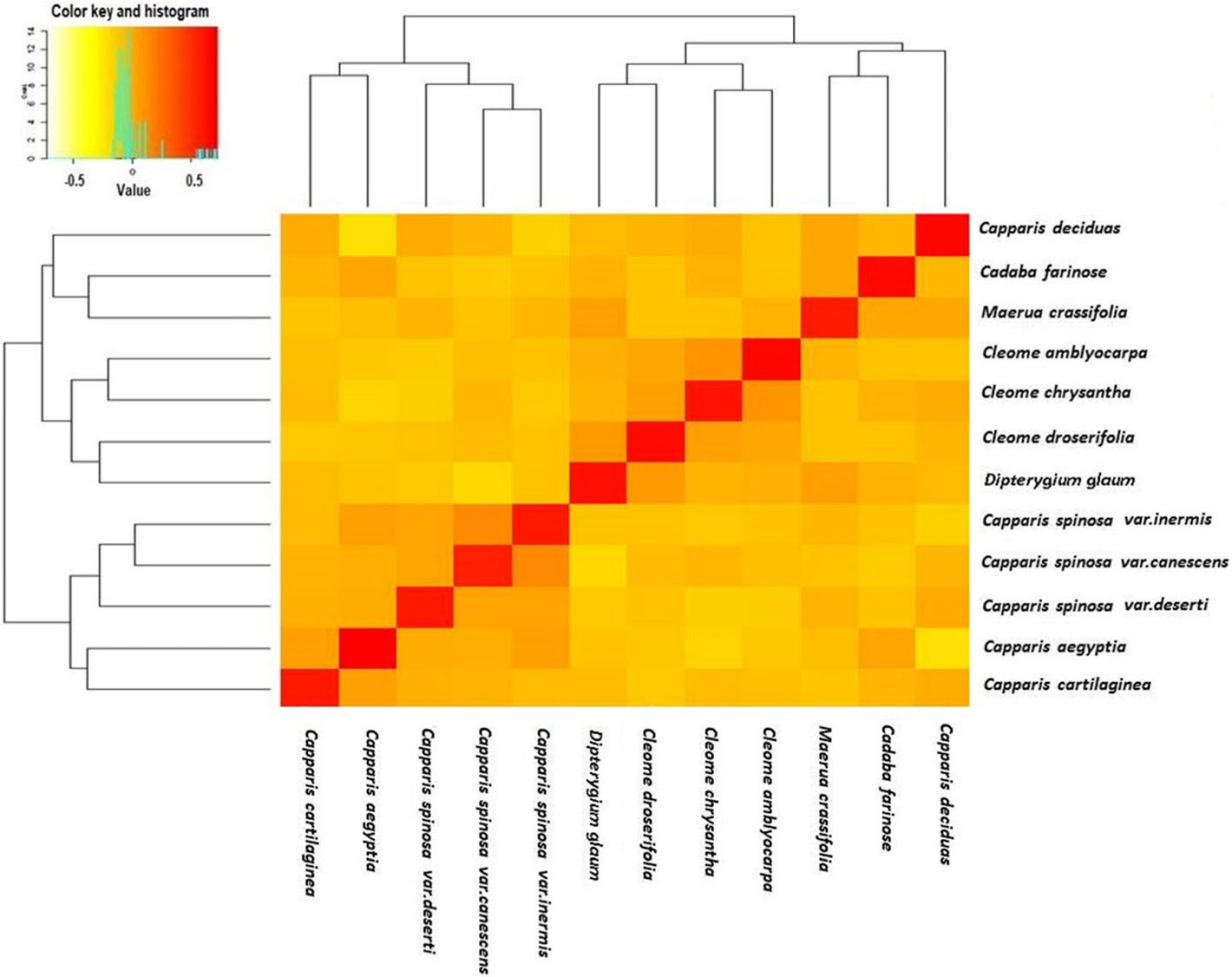
Heatmap cluster analysis (HCA) signatures among 12 *Capparaceae* species using ISTR profiles. Subclades are highlighted by a colorful background; the scale bar showed on the top illustrates the relative genetic variability from 0.5 to − 0.5

To further determine the genetic diversity, HCA exhibits the abundance of the relationships between the twelve species of *Capparaceae* and *Cleome*. The distribution of hot points indicates significant variations between the major groups of the *Capparaceae* and *Cleome* species and able to cluster in a sub-clade. As a result, the HCA was constructed based on the three sets of ISSR, SRAP, and ISTR markers (Fig. 7). The results were similar to each other with a tiny difference in the placement of some species, where the ISTR-HCA tree was the most consistent with the morphological taxonomy data of *Capparaceae*. Overall, three confirmed clades were identified, which have the ability to clearly distinguish among the twelve species. In detail, the first clade assembled together *Capparis decidua*, *Cadaba farinosa*, and *Maerua crassifolia* in a particular monophyletic clade. However, the three *Cleome* species and *Dipterygium glaucum* were placed jointly in the second clade with a high proportion of close relationships. In the framework, the third clade formed two monophyletic sub-clades, not based on their type but on their sampling origin, where the three species of *Capparis spinosa* are place jointly with high portions within the first sub-clade, whereas the second sub-clade occupies *Capparis aegyptia* and *Capparis cartilaginea* with a close genetic relationship. Collectively, we found both PCA and HCA data have drawn a successful annotation relationship in *Capparaceae* and *Cleome* species to evaluate whether the specific group sort individual or overlap groups.

## Discussion

The systematic approach and phylogenetic relationships of the Egyptian *Capparis* and *Cleome* species remain obscure, and unsolved dilemmas regarding their taxonomy and biology require further verifying and review [40]. Species identification in *Capparis* and *Cleome* is problematic because of the challenge of preserving the flowers [17] or maybe impossible when only vegetative parts are present, which is commonly the case during collection. Ettingshausen [41] made the primary comprehensive attempt to systematize the description of the vegetative leaf architecture together with his classification of venation patterns. Leaf architectural characters have demonstrated valuable taxonomic and systematic data both in fossil and living plants [42–44]. Leaf architecture and venation pattern were examined in numerous families of dicotyledons, among others, *Composite* [45], *Solanaceae* [46], *Bignoniaceae* [47], *Hamamelidaceae sensu lato* [48], *Leguminosae* [49], *Amaranthaceae* [50], *Ulmaceae* [51], *Fagaceae* [52], and in some monocots [53].

Various traditional taxonomic positions of *Capparis* and *Cleome* were derived from the study of both quantitative and qualitative macro-morphological characters which might become a broad margin of mistake. Therefore, additional knowledge about the genotype of plants is much required to resolve taxonomic problems in these genera [54]. Hence, evaluations of molecular statistics has had a serious effect on our perception of plant evolution and relations on all taxonomic levels, from the deep nodes dividing the key plant groups to species and populations [20]. Consequently, studying the levels of genetic diversity in natural populations is an essential precursor for the survey of plant species and will offer perceptions about the evolution of the species [55]. During the last two decades, there are little individual molecular studies involving *Capparis* and *Cleome* like the RAPD marker [54, 56] and therefore, the AFLP marker [57].

In the same context, the selection of the foremost appropriate marker system for a certain survey is not evident and principally depends on the aim of the research because the genetic structure of the species was varied [33]. The use of ISSR, SRAP, and ISTR marker is strongly recommended by several studies addressing the effectiveness of such markers for investigating *Capparaceae* diversity. One of the challenges of the current research is the use of ecological and molecular markers to explore the genetic relationships among the Egyptian *Capparis* and *Cleome* species grown within the Egyptian desert. In the present investigation, the relatively high values of the effective number of alleles for ISSR, SRAP, and ISTR markers were used to provide indication of their discrimination capacity when study a huge number of plants. This trend is required for the germplasm bank’s certification when multiple species require to be correctly distinguished and classified [58]. In this revised, the effective number of alleles tracking the method: SRAP > ISTR > ISSR. This result suggests that the ISTR and SRAP is more useful evidence for *Capparis* and *Cleome* species classification and certification. It is well-known that the marker index (MI) may probably be a suitable value for marker effectiveness [58]. By this criterion, arithmetically 1.18 fold greater MI was estimated for ISTR against SRAP and ISSR, highlighting the unique character of the ISTR assay. This is definitely owing to the superior value of effective multiples ratio (EMR) and assay efficiency index (Ai) [59]. Many studies confirmed that the retrotransposons marker, e.g., ISTR had a superior discrimination capacity and have the flexibility to detect several polymorphic loci per individual reaction [60]. Recently, Du et al. [61] suggested that retrotransposons (RT) occupied 28.1 Mb of the genome sequence, accounting for 9.74% of the entire genome. These results indicated that ISTR had an abundant presence of Ty-1 Copia retrotransposons, which permit obtaining useful polymorphism among the tested genotypes of *Capparaceae* and *Cleome* germplasm. Indeed, our finding showed that RT-ISTR markers had numerous unique private loci that would allow diversity within the sub-species of *Capparaceae* and *Cleome* germplasm, which is in concurrence with earlier reports of this marker [31, 62].

In the Mediterranean area, five species are confirmed to be growing, e.g., *Capparis aegyptia* Lam. Boiss., *Capparis spinosa* L., *Capparis orientalis* Veil., *Capparis sicula* Veil., and *Capparis ovata* Desf [63, 64]. Mainly, in various floristic works, *Capparis aegyptia* is recognized from Northern Africa and also the Mideast [65]. In Flora Hellenica [66], it has been raised, *Capparis aegyptia* Lam., to a subspecies of *Capparis spinosa*, while Inocencio et al. [63] returned its status of individual species, while Özbek and Kara, [67] fully proposed that the two subspecies *Capparis spinosa* L. and *Capparis ovata* Desf. might be distinguished roughly. However, Inocencio et al. [63] confirm that *Capparis aegyptia* Lam. and *Capparis ovata* are very distinct from the opposite taxa but extremely tight to each other.

Recently, Al-Safadi et al. [64] recognized three *Capparis* species growing in Syria, *C. sicula Duh*, *C. aegyptia* Lam, and *C. spinosa* L., and the results support this theory that *C. aegyptia* Lam. might be an independent species and not a varietal level of *C. spinosa*. Initially, Rivera et al. [68] discussed the origin of *Capparis cartilaginea* and indicated that this is often a widely accepted name. Following the phylogenetic analysis of Saifi et al. [69], the dendrogram was assembled together with *Capparis cartilaginea*, *Capparis aegyptia* Lam, and *Capparis spinosa* in a sub-group. In view of the previous revisions, our phylogenetic analysis clear evidence supporting the undisputed viewpoint that *Capparis aegyptia* and *Capparis cartilaginea* are very closely associated with *Capparis spinosa* in a sister clade and seem to be distinguished. These results might be applied in systematics and evolutionary biology studies within the Egyptian species of *Capparis* and *Cleome* to clarify the complex interactions among species, as demonstrated in previous studies [64, 70]. Indeed, we are able to tentatively imply forward this theory as *Capparis decidua*, *Cadaba farinosa*, *Maerua crassifolia*, and *Dipterygium glaucum* formed a particular monophyletic clade with *Cleome* species. Based on the above considerations, our results verified the conclusions of systematic and taxonomic analyses that were performed on the collected samples, which is in consensus with previous articles on *Capparis* species [54, 57]. Interestingly, we observed a thorny interspecies in *Capparis decidua*, *Cadaba farinosa*, *Maerua crassifolia*, and *Dipterygium glaucum* specimens and also the concerned during this study. Currently, the advent of molecular markers overcame the majority of the challenges related to utilizing morphological markers during which main phenotype-varying genes were applied as genetic markers [71].

## Conclusion

In the present investigation, we emphasize that the outcomes of the morphological and ecological characterization as well as the genetic analysis based ISSR, SRAP, and SRAP can capture the taxonomy and systematics of the various subgroups recovered with a good performance in clarifying genetic diversity within and among populations in the Egyptian *Capparis* and *Cleome* species. Our findings show *Cleome* and *Gynandropsis* genera as an explicit family; therefore, an in-depth study like next-generation sequencing (NGS) technologies is now emerging as precision tools to assess the molecular systematics and evolution in *Dipterygium glaucum* (if belongs to *Capparaceae* or *Cleomaceae* or another family). Collectively our results not only help within the classification of species but may distinguish species limitations, flagging of modern species, and genus delimitation.

## Data Availability

The datasets used and/or analyzed during the current study are available from the corresponding author on reasonable request.

## Abbreviations

ISSRs: Inter-simple sequence repeats
SRAP: Sequence-related amplified polymorphism
ORFs: Open reading frames
ISTR: Inverse sequence-tagged repeat
TWINSPAN: A hierarchical dichotomous analysis
PIC: Polymorphic information content
MI: Marker index
PCA: Principal coordinates analysis
HCA: Heatmap cluster analysis
